# Electrolyte changes: An indirect method to assess irrigation fluid absorption complications during transurethral resection of prostate: A prospective study

**DOI:** 10.4103/1658-354X.71505

**Published:** 2010

**Authors:** Kumkum Gupta, Bhawna Rastogi, Manish Jain, Prashant K. Gupta, Deepak Sharma

**Affiliations:** *Department of Anesthesiology & Critical Care, N.S.C.B.Subharti Medical College, Subhartipuram, NH-58, Meerut - 250 004, U.P. India*; 1 *Department of Radio-diagnosis, Imaging & Interventional Radiology, N.S.C.B.Subharti Medical College, Subhartipuram, NH-58, Meerut - 250 004, U.P. India*

**Keywords:** *Benign prostatic hyperplasia*, *transurethral resection of prostate syndrome*, *1.5% glycine*, *hyponatremia, hyperkalemia*, *central neuraxial block*

## Abstract

**Context:**

Fluid absorption is inevitable complication of transuretheral resection of prostate and serum electrolytes changes can indirectly assess the irrigation fluid absorption.

**Aims:**

To monitor the extent of 1.5% glycineirrigation fluid absorption during transurethral resection of prostate (TURP), by measuring the changes of serum sodium and potassium levels peri-operatively.

**Settings and Design:**

This is a randomized prospective cohort observational study.

**Materials and Methods:**

The 86 male patients of ASA grades I to III in the age group of 50 to 80 years, scheduled for elective TURP surgery under central neuraxial block, were studied. Their preoperative and post-operative serum sodium, potassium and calcium levels were measured. When duration of surgery exceeds 60 min, serum sodium and potassium levels were done intra-operatively with venous blood samples by using blood gas analyser. The height of irrigation fluid column was kept constant at 60 cm. These changes were correlated with the volume of irrigating fluid used, duration of procedure and the volume of prostate gland resected.

**Statistical Analysis used:**

The values of pre and postoperative sodium, potassium and calcium serum levels were compared and statistical significance of the difference in values was assessed using Student’s paired *t* test.

**Results:**

Statistically significant reduction of serum sodium levels (hyponatremia) and elevation of serum potassium levels (hyperkalemia) were observed post-operatively, which was directly proportional to volume of irrigating fluid used, duration of procedure and volume of prostate gland resected. No significant changes in serum calcium level were observed.

**Conclusions:**

To measure serum electrolytes changes during TURP surgery, it is simple and economical method for indirect assessment of fluid absorption for early identification of TURP syndrome.

## INTRODUCTION

Benign prostatic hyperplasia (BPH) is common worldwide in men over 40 years of age. The open prostatectomy and transurethral resection of prostate (TURP) have been the surgical options for men with obstructive symptoms. TURP is the second most common surgical procedure done in men. It requires the use of irrigating fluid to gently dilate the mucosal spaces, remove blood, cut tissue and debris from the operating field and enable better vision. Various irrigating fluids have been used for TURP.[1] The potential complication of such procedure is systemic absorption of hypotonic irrigating fluid.

The various clinical manifestations, produced due to the absorption of large volume of irrigating fluid during transurethral resection of prostate, are together known as TURP syndrome. Early signs of TURP syndrome are dizziness, headache, nausea, dyspnea, arrhythmias, hypertension and bradycardia, followed by restlessness and confusion. If not treated promptly, a patient becomes cyanotic, hypotensive and ultimately sustains cardiac arrest. Occasionally, it starts with neurological signs.[2] These symptoms primarily are manifestations of circulatory fluid overload, water intoxication and occasionally toxicity of the solute in the irrigating fluid. Despite improvements in the current surgical and anesthetic management, 2.5-20% of patients undergoing TURP show one or more manifestations of TURP syndrome and 0.5-5% die perioperatively.

### Pathophysiology of TURP syndrome

Hahn *et al*. postulated in 1990[3] that water intoxication with hyponatremia is the primary cause for the genesis of TURP syndrome. Irrigating fluid is directly absorbed into the vascular system when prostatic tissue has been cut along with vein by electrocautery. The driving force is the fluid pressure which needs to exceed venous pressure of 1.5 kPa.[4] The amount of fluid absorbed via the prostatic veins is 20 mL/min, and can reach up to several liters. The uptake of 1 L of fluid within1 h corresponds to an acute decrease in the serum sodium concentration of 5-8 mmols/L. For every 100 mL of fluid entering the interstitial compartment, 10-20 mEq of sodium also moves with it.[5] Symptoms of hyponatremia usually do not develop until the serum sodium concentration decreases below 120 mEq/L. The osmolality of 1.5% glycine is 230 mOsm/L(hypotonic) as compared to serum osmolality of 290 mOsm/L. This marked hypotonicity in plasma may also result in intravascular hemolysis which leads to elevation of serum potassium level. Clinical manifestations and ECG changes (peaked T waves, prolongation of QRS complex and PR interval and ventricular fibrillation) of hyperkalemia occur when the plasma level of potassium rise above 6 mEq/L. Hyperkalemic cardiotoxicity is increased by hyponatremia and acidosis.[6] Therefore it is possible that the cardiovascular changes occurring in TURP syndrome may be due to the combination of hyponatremia and hyperkalemia. Hahn *et al*. also found significant elevation of serum potassium during absorption of irrigating fluid intraoperatively. Absorption in excess of 1 L of glycine solution, is associated with the increased risk of symptoms which appeared 30-45 min, after surgery is completed. Visual disturbances such as blurred vision, transient blindness, and pupillary dilation have been reported with TURP syndrome. There are clinical reports of transient blindness after absorption of 1.5% glycine which resolves within 24 h.[7] Different methods for assessing the fluid and electrolyte imbalance during TURP have been suggested which include the use of load cell transducers,[8] measurement of serum acid phosphatise,[9] assessment of breath ethanol,[10] and monitoring of plasma concentration of fluorescein.[11] Smoking is the only known risk factor, associated with fluid absorption during TURP as demonstrated by Hahn RG in 2001.[12]

In our study, we have analyzed the changes in serum electrolytes with use of 1.5% glycine solution as irrigating fluid, during TURP (transurethral resection of prostate), which help to recognize fluid absorption and hence early development of TURP syndrome. The changes in serum electrolytes also have been correlated with duration of the procedure, volume of 1.5% glycine used and volume of prostate gland resected.

## MATERIALS AND METHODS

This prospective study was conducted on 86 adult consented male patients with ASA grades I to III in the age group of 50 to 80 years, scheduled for elective TURP under central neuraxial block, from July 2008 to July 2009.

Clearance from Ethical Committee of Institution was obtained. All patients were subjected to pre-anesthetic assessment prior to TURP surgery. They were assessed with routine investigations for geriatric anesthesia, including hematocrit, ECG, Doppler echocardiography, preoperative serum sodium, potassium and calcium levels (in mEq/L) and fundus examination. If needed, the patients were optimized. Patients with history of smoking, pre-existing cardiac or pulmonary diseases, and renal dysfunction were excluded from study. Metastasis in the lumber spine, a contraindication to central neuraxial block, was also the exclusion criteria.

All patients were preloaded with 10 mL/kg ringer lactate solution and standard monitors for heart rate, systemic blood pressure, ECG, SpO_2_ were attached. Central neuraxial block was performed aseptically at L2-3 or L3-4 intervertebral disk space in sitting posture and without difficulty producing satisfactory analgesia to a dermatome level up to T10. Patients were positioned in lithotomy posture and the TURP surgery was started with warm 1.5% glycineirrigation fluid, keeping the irrigation fluid column at a height of 60 cm, measured from the level of pubic symphysis of the patients on the operating table. The duration of procedure in minutes, volume of prostate gland resected, and the volume of 1.5% glycine used during the procedure were recorded. Intraoperatively, the serum sodium and serum potassium levels were done in patients undergoing surgery over 60 min by blood gas analyzer (Osmetach OPTI, CCA-TS) by using venous blood samples. All patients were carefully observed for the early symptoms of TURP syndrome perioperatively. The procedure was terminated when serum sodium level was <125 mEq/L, serum potassium level was >6.0 mEq/L or early signs of restlessness, bradycardia, yawning, etc. have occurred. Adequate therapeutic measures were taken to prevent further complications.

The serum levels of sodium, potassium, and calcium were also measured after 1 h post-operatively. The values of pre and post-operative serum sodium, potassium and calcium levels were compared and statistical significance of the difference in values was assessed using Student’s paired t test. The changes in the plasma electrolytes levels were also correlated with the volume of irrigating fluid used, duration of procedure, and the volume of prostate gland resected.

## RESULTS

All the 86 patients underwent TURP surgery using 1.5% glycine as irrigating fluid. The volume of the prostate gland resected ranged from 30 mL to 80 mL. The duration of the TURP surgery ranged from 45 to 90 min and the volume of the 1.5% glycine used, ranged from 3 to12 L.

In our study, clinical manifestation of full blown TURP syndrome was not seen due to precautions been taken intraoperatively. Seven patients (8.13%) showed restlessness and bradycardia when duration of TURP exceeded 60 min which corresponded with decreasing serum sodium and increasing serum potassium levels, done intraoperatively with venous blood samples using blood gas analyzer.

The mean levels of serum sodium and potassium pre and post-operatively in patients undergoing TURP surgery are given in Tables 1 and 2.

**Table 1:** Changes in serum level of sodium during TURP

Duration	45-55 min		55-65 min		65-85 min	
Volume of irrigation fluid used	3-5 L		6-10 L		11-12 L	
Volume of prostate	30-50 mL		51-60 mL		61-80 mL	
	
	**Preop**	**Postop**	**Preop**	**Postop**	**Preop**	**Postop**
	
Sodium (mEq/L)mean	141.84+ 2.44	140.03+ 2.56	141.39+2.56	136.52+ 2.61	141+2.70	130.36+ 2.73
% change		1.27%		3.4%		7.5%
*P*-value		.003		0.000		0.000

*P-value<0.05 is significant **P-value<0.001 is highly significant; TURP: Transurethral resection of prostate

**Table 2:** Changes in serum potassium level during TURP

Duration	45-55 min		55-65 min		65-85 min	
Volume of irrigation fluid used	3-5 L		6-10 L		11-12 L	
Volume of prostate	30-50 mL		51-60 mL		61-80 mL	
	
	**Preop**	**Postop**	**Preop**	**Postop**	**Preop**	**Postop**
	
Potassium (mEq/L)mean	4.33+ 0.56	4.55+0.57	4.46+0.50	4.96+0.48	4.26+0.57	4.95+0.56
% change		5.0%		11.21%		16.19%
*P*-value		.004		0.000		0.000

*P-value<0.05 is significant **P-value<0.001 is highly significant; TURP: Transurethral resection of prostate

The mean level of serum sodium showed statistically significant reduction (hyponatremia) post-operatively during surgical procedure. There was statistically significant increase in the mean level of serum potassium (hyperkalemia) post-operatively. The percent rise in potassium levels (5-11.21%) was much higher as compared to the percentage change in sodium levels (1.27-3.4%) during the TURP surgery. There was no significant change in mean levels of serum calcium post-operatively.

The changes of serum sodium and potassium levels were directly proportional with volume of 1.5% glycine used for irrigation, duration of TURP and the volume of prostate gland resected [Tables 1 and 2].

## DISCUSSION

Transuretheral resection of the prostate is considered by many as simpler and safer procedure than open prostatectomy. Patients who have undergone TURP are often elderly and suffer from cardiac, pulmonary, renal and endocrine disorders. Occasionally, these patients are dehydrated and depleted of essential electrolytes e.g. sodium, calcium and potassium because of long-term diuretics along with restricted fluid intake. Endourological surgery is associated with intraoperative complications of bleeding and deleterious systemic effects due to intravesical absorption of irrigating fluid. Water intoxication with hyponatremia has been postulated by Hahn *et al*. in 1990 as the primary cause for the genesis of TURP syndrome.[3] TURP syndrome is characterized by intravascular volume shifts and plasma-solute (osmolarity) effects. Absorption of small amounts of fluid occurs with dilutional hyponatraemia and patients begins to complain of dizziness, headaches, nausea, dysnea, etc. However, other adverse effects due to more fluid absorption soon became apparent. Hyponatraemia may cause weakness, muscular twitches and epileptic seizures. Severe events are associated with absorption of >3 L of fluid.

In our study, we have also found the significant changes in serum sodium levels (hyponatremia) with the change of volume of 1.5% glycine used, duration of procedure and with volume of prostate gland resected.

Serum potassium often increases transiently by 15-25% in response to 1.5% glycineirrigation fluid absorption, probably related to intracellular uptake of the irrigant solute. Clinical manifestations of hyperkalemia occur when the plasma levels of potassium rise above 6 mEq/L along with ECG changes of tall, peaked T waves, PR prolongation, QRS widening and arrhythmias. Hyperkalemic cardiotoxicity is increased by hyponatremia and acidosis. These cardiotoxic effects of hyperkalemia, occur during TURP surgery, will be less marked if serum calcium levels are within normal limits of 8.5-10.5 mg/dL (2.1-2.6 mmol/L).

Our study also showed that significant hyperkalemia occurred during TURP which was dependent more on the duration of procedure rather than on other determinants. Hahn *et al*. also found significant elevation of serum potassium during absorption of 1.5% glycine solution into circulation intra-operatively. The cause of hyperkalemia was inability of 1.5% glycine to maintain the isotonicity of plasma.[13,14]

Madsen *et al*. in 1973 demonstrated one another important factor which would also determine the rate of fluid absorption which will be the hydrostatic pressure at the prostatic bed, depending on the height of irrigating fluid column and the pressure inside the bladder during surgery. The ideal height of irrigating fluid is 60 cm so that approximately 300 mL of fluid is obtained per minute during resection for good vision.[15] For this study, we have fixed the irrigation fluid column height at 60 cm, so that other determinants can be assessed for changes in serum electrolytes during TURP surgery.

The regional technique of central neuraxial block was preferred over general anesthesia as it allowed observation of early symptoms and signs of TURP syndrome. Regional anesthesia may reduce the incidence of post-operative venous thrombosis. Clinical studies have failed to show any differences in blood loss, post-operative cognitive function and mortality between regional and general anesthesia.[16]

Blood loss was difficult to assess because of use of irrigating solutions, so it was necessary to rely on clinical signs of hypovolumia. Blood loss averages about 3-5 mL/min of resection, usually 200-300 mL and was rarely life threatening.

Irrigation fluid was warmed to body temperature to prevent hypothermia during TURP.

Electrolytes solutions cannot be used for irrigation during TURP surgery as they disperse the electrocautery current. Water provides better visibility due to hemolysis but significant absorption will cause acute water intoxication. The 1.5% glycine, an endogenous amino acid has been suggested as a suitable irrigating fluid considering its many advantages, including the low cost, its tendency to cause less hemolysis and renal failure. Glycine 2.2% is isotonic with plasma but the side effects of glycine at this concentration are more. Transient blindness can also occur after absorption of the glycine, as it is an inhibitory neurotransmitter in the retina. The mixture of sorbitol 2.7% and mannitol 0.54% (195 mOsm/L) has also been used. Less commonly used solutions include sorbitol 3.3%, mannitol 3%, dextrose 2.5-4%, and urea 1%. All these fluids are hypotonic so significant water absorption can occur. Solute absorption can also occur when the irrigation fluid is under pressure. The use of large amounts of sorbitol or dextrose irrigating solutions can lead to hyperglycemia, which can be marked in diabetic patients. Absorption of mannitol 3% solution causes intravascular volume expansion more than 1.5% glycine, while sorbitol–mannitol takes an intermediate position.[17] The morbidity and mortality were found to be definitely higher when surgery was prolonged over 90 min.

Several methods have been proposed to reduce the risk of fluid absorption but none is capable of eliminating its complication. Monitoring the extent of fluid absorption during surgery allows control of the fluid balance in every patient. The most viable methods to monitor fluid absorption are ethanol monitoring and gravimetric weighing.[18] Newer techniques, such as bipolar resectoscopes and vaporizing the tissue instead of resecting tissue, would reduce the fluid absorption and its consequences.[19] Reuter *et al*. demonstrated in 1978 that low pressure irrigation during TURP would limit the risk of intravascular absorption.[20] The surgeon should be notified about ongoing fluid absorption whenever it exceeds 1 L. This allows steps to be taken to prevent excessive absorption.

Supportive care remains the most important therapeutic approach for management of complications. Treatment for dilution hyponatremia should be based on administration of hypertonic saline rather than on diuretics. Administration of calcium, alkalinisation and even hemodialysis in severe cases can counter the potassium toxicity.[21]

## CONCLUSION

The pathophysiological mechanism of TURP syndrome consists of pharmacological effects of irrigant solute, volume effects of irrigant fluid and serum electolytes changes, so it is very difficult to avoid the occurrence of TURP syndrome. The best prevention method could be obtained by adopting a correct surgical technique and optimizing the patients conditions pre-operatively. Therefore our study emphasized the need to assess serum levels of sodium, potassium and calcium pre-operatively in patients scheduled for TURP (transurethral resection of prostate) surgery and optimization of serum electrolytes to prevent serious and fatal complications. Normally the fall in serum sodium during TURP has been identified to be in the range of 5-8 mEq/L. However the cardiotoxic effects of hyponatremia will be dependent on the concomitant variation in serum potassium levels during the procedure. Procedure lasting for more than 60 min and volume of prostate gland more than 60 mL could be associated with more complications. Limitation of the height of irrigating fluid column to 60 cm could provide optimum vision to the surgeon and reduce the complications of fluid absorption. Glycine 1.5%, although slightly hypotonic, is widely used for irrigation as it has good optical properties, is nonelectrolytic and prevents dissipation of diathermy current during resection. Early identification of TURP syndrome and its treatment should be based on administration of hypertonic saline.

Laser TURP can help to minimize fluid absorption and its complications especially in cardiac and critically ill patients.
